# Coordination between Rac1 and Rab Proteins: Functional Implications in Health and Disease

**DOI:** 10.3390/cells8050396

**Published:** 2019-04-29

**Authors:** Azzurra Margiotta, Cecilia Bucci

**Affiliations:** 1 Centre for Immune Regulation, Department of Biosciences, University of Oslo, 0371 Oslo, Norway; 2 Department of Biological and Environmental Sciences and Technologies, University of Salento, 73100 Lecce, Italy

**Keywords:** Rac1, Rab proteins, actin cytoskeleton, GTPase, Rho proteins

## Abstract

The small GTPases of the Rho family regulate many aspects of actin dynamics, but are functionally connected to many other cellular processes. Rac1, a member of this family, besides its known function in the regulation of actin cytoskeleton, plays a key role in the production of reactive oxygen species, in gene transcription, in DNA repair, and also has been proven to have specific roles in neurons. This review focuses on the cooperation between Rac1 and Rab proteins, analyzing how the coordination between these GTPases impact on cells and how alterations of their functions lead to disease.

## 1. Introduction

Ras-related C3 botulinum toxin substrate 1 (Rac1) is a member of the family of the typical Rho guanosine triphosphate phosphohydrolases (GTPases), which are known for their role in several cellular processes such as cytoskeleton organization, gene expression regulation, and cell migration [1]. Rac1 is one of the best-characterized typical Rho GTPases, and as all the small G proteins belonging to this group, it can cycle between an active guanosine triphosphate (GTP)-bound and an inactive guanosine diphosphate (GDP)-bound conformation. Modulation of Rac1 activity is highly regulated by several proteins, affecting its localization, expression levels, and functions [1]. Indeed, replacement of GDP with GTP is favored by guanine nucleotide exchange factors (GEFs) that lead to Rac1 activation, while its inactivation is regulated by GTPase activating proteins (GAPs) that promote GTP hydrolysis [1]. Guanine nucleotide dissociation inhibitors (GDIs) are instead responsible for controlling the GTPase activity by extracting it from the membranes where it exerts its role and holding it in an inactive state in the cytosol [1]. Rac1 is a signaling G protein that regulates a number of cellular events, among which, besides cytoskeletal reorganization, there are cell growth and activation of kinases. Rac1 signaling is also modulated through post-translational modifications [2,3,4,5,6,7,8,9] (Table 1). In fact, Rac1 is prenylated and subsequently palmitoylated at Cys178, and these post-translational modifications affect Rac1 localization and activity [2]. Furthermore, several studies have shown that active Rac1 bound to the plasma membrane can be ubiquitinated and subjected to proteasome-mediated degradation, leading to disassembly of epithelial cell-cell contacts and to a regulatory feedback on its activity [3,4]. The major target site for Rac1 ubiquitination is Lys147 and seems to be dependent on JNK (Jun N-terminal Kinase) activation [4], although also the tumor suppressor HACE1 has a role in the regulation of Rac1 ubiquitylation and activity [5,9]. Among the Rac1 post-translational modifications, there is SUMOylation by a small ubiquitin-like protein (SUMO) that is covalently linked to lysine residues. SUMOylation of the C-terminal domain of Rac1 leads to increased activity of this GTPase, and it is mediated by the SUMO E3-ligase PIAS3, which, interacting with Rac1, regulates cell migration [6]. Finally, Rac1 is also phosphorylated on Ser71 by Akt and on Thr108 by ERK [7,8]. Phosphorylation of Ser71 seems to inhibit Rac1 GTP-binding activity without affecting its GTPase activity [7]. Modulation of the phosphorylation of this site is responsible for alterations of selected downstream pathways and specific cell functions, such as, for instance, membrane ruffling and PAK1/2 activation [10]. Phosphorylation of Thr108 inhibits Rac1 interaction with the phospholipase C-γ1 (PLC-γ1) and Rac1 activity, leading to its translocation to the nucleus, affecting in turn cell migration [8]. Besides to the plasma membrane and the nucleus, Rac1 can be recruited also to the mitochondria, where, in its active form, it is responsible for the H_2_O_2_ production in macrophages [11,12]. 

Active Rac1 can interact with a multitude of effector proteins that confer to this small GTPase the ability to mediate several cellular processes that are essential for physiological cell functions [13,14,15,16,17,18]. The main role of Rac1 is the regulation of actin cytoskeleton [19,20]. In fact, this small GTPase is implicated in controlling the formation of membrane ruffles, lamellipodia, actin stress fibers, and focal adhesion [19,20,21]. However, it has been proven that Rac1 is important also in gene expression, cellular plasticity, production of reactive oxygen species (ROS), cellular adhesions, migration and invasion, cell proliferation, apoptosis, and inflammatory responses [22,23,24,25,26,27,28,29,30,31,32,33,34,35,36,37,38,39,40,41]. As Rac1 exerts several physiological functions, alterations of its signaling are implicated in many diseases, such as cancer, cardiovascular disease, arthritis, kidney disorders, pathological inflammatory responses, infectious diseases, and neurodegenerative disorders [38,39,40,41,42,43,44,45,46,47,48,49,50,51,52].

Interestingly, several studies have proven connections between proteins belonging to the Rho family and proteins from another family of small GTPases, the Rab proteins [53,54]. These discovered connections revealed that alteration of coordination between Rho proteins, and in particular Rac1, and Rabs has important implications in several human diseases.

## 2. Rac1 and Rabs: Interactions and Regulations of Their Functions

Physical interactions between Rac1 and members of the Rab family have been reported [55]. In fact, Rac1 binds strongly to Rab7a wt and to the Rab7a Q67L constitutively-active mutant, but very weakly to the Rab7a T22N dominant negative mutant, indicating that Rac1 binds preferentially to the GTP-bound form of Rab7a [55]. Interestingly, the GTP-bound form of Rac1 affects Rab7a activity also through one of its effectors, called Armus, which is a Rab7a GAP [17]. Furthermore, it has also been proven that Rab7a is able to regulate Rac1 activity, although the molecular mechanism is not known yet [56,57]. These data suggest that the coordination between Rac1 and Rab proteins is accomplished at different levels in order to control finely their functions. 

Furthermore, Rab23 has been proven to affect Rac1 by regulating a Rac1 GEF, Tiam1 [58]. Indeed, GTP-bound Rab23 interacts with β1 integrin, which then recruits Tiam1 and activates Rac1 [58]. Binding of Rab23 to Tiam1 is dependent on the presence of β1 integrin [58]. Furthermore, Rab5 activity is regulated by Rac1 through a GEF [59]. In fact, active Rac1 interacts with alsin, a Rab5 GEF, and a Rac1 effector and activates the Rab GTPase [59]. All together, these data demonstrate that one important level of regulation is based on modulating the activity of GTPase modulators such as GEFs and GAPs.

So far, only a few direct interactions between Rac1 and Rab proteins and/or their regulators have been discovered, but clearly, this could be a general mechanism for their coordination responsible for cellular altered functions and possibly diseases.

## 3. Rac1 in Cancer: Role of Rac1 and Rabs in Cell Migration and Metastasis

Cancer is a complex group of diseases characterized by uncontrolled cell growth. Excess of Rac1 activity has been linked to several cancer types such as breast cancer, colorectal cancer, gastric cancer, prostate cancer, and cervical cancer [60,61,62,63,64,65]. In particular, Rac1 is involved in several phases of cancer development, as it can promote cancer initiation, cancer progression, and metastases through its role in gene transcription, cell cycle progression, neovascularization, cell adhesion, migration, and invasion [56,66,67,68,69]. Moreover, mutations of Rac1 could determine a pathological state as Rac1 stimulates macropinocytosis, a fluid-phase endocytosis driven by actin-based protrusion of the plasma membrane [19]. Macropinocytosis relies on large organelles called macropinosomes that allow internalization of extracellular material that it is subsequently used by cancer cells to increase their metabolism, therefore contributing to the growth of the tumor [70].

The most important feature of cancer, negatively related to rate of survival, is the ability of cancer cells to spread to other parts of the body through a process called metastasis, which is characterized by cell migration and invasion. Cell migration is a process that consists of the formation of cell protrusions such as lamellipodia and filopodia and new adhesion sites at the front of the cell (leading edge), contraction of the cell body, and detachment of adhesions at the rear [71,72]. Several studies have demonstrated that Rac1 is involved in the regulation of cell migration and invasion and that Rab proteins can collaborate in this process [17,56,58,73,74,75,76,77,78,79].

**Figure 1:**
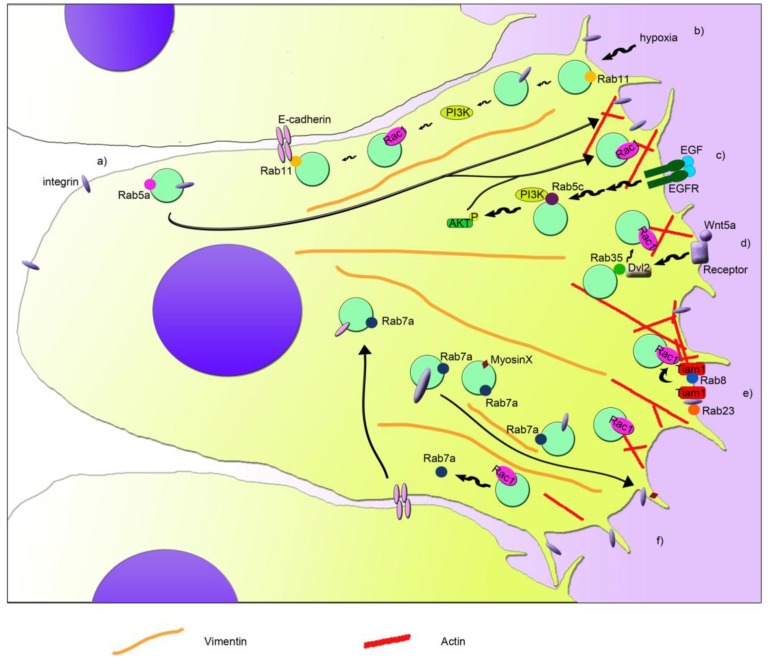
Schematic model that depicts the coordination of Rab proteins and Rac1 in cell migration. Different pathways are indicated with letters. (**a**) Rab5a regulates the internalization and recycling of integrins from the rear edge to the leading edge. This regulation is important for the formation of new adhesions at the front of the cell, for Rac1 activation, and for actin filament (in red) reorganization. (**b**) Rab11 interacts with E-cadherin and recruits and activates Rac1 at the plasma membrane. In hypoxia, αvβ3 integrin and PI3K are activated through the action of Rab11, leading to Rac1 activation and cell migration. (**c**) Cell stimulation with EGF and its binding to EGFR modulates activity of PI3K and AKT in a Rab5c-mediated manner. This in turn leads to increased Rac1 activity, membrane ruffles, and cell migration. (**d**) Wnt5a signaling induces the activation of Dvl2, which binds to Rab35 activating it. Active Rab35 increases Rac1 activity and therefore cell migration. (**e**) Rab8 activation induces Rac1 activation mediated by a Rac1 GEF called Tiam1, regulating cortical actin polymerization and focal adhesion reorganization. Furthermore, active Rab23 interacts with Tiam1, through its interaction with β1 integrin, regulating Rac1 activity and cell migration. (**f**) Rab7a regulates cell migration by modulating Rac1 activity, β1 integrin, and myosin X transport at the leading edge and the regulation of vimentin filament organization (in orange). Moreover, Rac1 regulates Rab7a activity and in turn E-cadherin degradation in lysosomes, and therefore cell-cell contacts.

Among Rab proteins, Rab5a is responsible for the regulation of the trafficking between the plasma membrane and early endosomes [80,81]. However, Rab5a is required also for the activation of Rac1 and, in turn, for the regulation of the actin cytoskeletal organization [82] (Figure 1a). In particular, Rab5a is able to regulate the formation of integrin and adhesion complexes and, in turn, controls Rac1 activity and the organization of actin structures [73]. Therefore, Rab5a silencing reduces the number and size of protrusions and decreases cancer cell motility and invasion, which is important for metastasis and tumor spread [73]. Another isoform of Rab5, Rab5c, has been linked to the regulation of Rac1 activity dependent on EGF and therefore to cell migration [74]. Similar to Rab5a, Rab5c depletion determines the presence of fewer focal adhesion foci, less membrane ruffles, and less cell migration [74]. In fact, Rab5c is responsible for recruiting Rac1 at the plasma membrane, where it promotes the formation of lamellipodia [74]. In line with this, Rab5c silencing affects the abundance of Rac1 in the membrane fraction when compared to control cells [74]. Moreover, upon stimulation with EGF, Rab5c-depleted cells show less phosphorylated, active, AKT and, in turn, PI3 kinases [74]. This leads to a reduction of EGF-stimulated Rac1 activity in Rab5c-depleted cells compared to control cells, although lower Rac1 activity has been detected also at steady state upon Rab5c silencing [74] (Figure 1c). 

Interestingly, also the interplay between Rac1 and Rab7a, which is localized mainly to late endosomes and regulates the late steps of endocytosis [83,84,85], has an important role in cell migration and metastasis [17,55,56]. Indeed, Rab7a interacts with Rac1 [55], and they can affect each other’s activation state [17,56]. Depletion of Rab7a induces a lower activation of Rac1, which in coordination with other effects on the activation of β1 integrin, organization of vimentin filament, and trafficking of myosin X, determines a negative effect on migration, formation of protrusions, adhesion, and spreading [56], similarly to Rab5a and Rab5c (Figure 1f). On the contrary, Rac1 activation determines a lower amount of active Rab7a by acting on Rac1-effector armus, which is a bona fide GAP for Rab7a [17]. 

The coordination of Rac1 and Rab7a in epithelial cells is important for cell-cell adhesion and for collective cell migration, epithelial-mesenchymal transition (EMT), and metastasis. Integrated signaling among Arf6, Rac1, and Rab7a is necessary for regulating E-cadherin degradation, which in turn, leads to the loss of cell-cell contacts [17]. Arf6 is able to activate both Rac1 and Rab7a, leading to internalization of E-cadherin, which becomes mainly perinuclear. Nevertheless, active Rac1 is able to regulate Rab7a’s activation state through its effector armus, which inhibits Rab7a, blocking E-cadherin degradation in lysosomes [17]. Modulation of E-cadherin surface levels is fundamental for cell adhesion of epithelia both in health and disease, affecting epithelial morphogenesis and differentiation, but also acquisition of mesenchymal characteristics. Epithelial cancer cells can detach from the close layer of cells, undergo EMT, and spread in other tissues, becoming a metastatic cell. Interestingly, upregulation of Rab7a and armus-related proteins has been reported in several epithelial tumors [17,86,87]. In line with the effect of active Rac1 on the activation state of Rab7a, less Rab7a is pulled down by RILP, a GTP-Rab7a interactor [88], when Rac1 is activated [17]. We recently proved that also RILP is able to regulate cell adhesion and migration [89]. Although Rab5a, Rab5c, or Rab7a silencing showed a reduction in cell migration [56,73,74], RILP depletion determined an increase in cell motility and speed [89]. Interestingly, RILP-depleted cells showed the ability to migrate as single cells [89], prompting the evaluation of E-cadherin turnover in these cells, in order to elucidate the mechanisms behind the regulation of cell migration and adhesion mediated by Rab7a and RILP.

Another connection between Rac1 and Rab GTPases in the regulation of cell migration is the one discovered between Rab8 and Rac1 [75]. Rab8 is important in membrane transport to the plasma membrane, but it also regulates the organization of the actin cytoskeleton and the size and distribution of focal adhesions during cell migration [75,90,91,92]. Interestingly, the Rab8-dependent regulation of the actin cytoskeleton organization is mediated by Rac1. In fact, expression of a constitutively-active mutant of Rab8 induced an increase of Rac1 activity, probably due to the redistribution of Tiam1, a Rac1 GEF, to the plasma membrane (Figure 1e). This coordination regulates cell protrusions and contributes to the loss of focal adhesions [75]. Surprisingly, Tiam1-dependent activation has been observed also in Rab5-positive endosomes for the regulation of actin polymerization in dorsal ruffles [93]. However, specific recruitment of Rab8 to the protrusive edge is necessary to ensure persistent migration, and it could be mediated by the Rab8 GEF Rabin8, a Rab11 effector responsible for the apical exocytosis during lumen formation mediated by Rab8 [75,94]. Similar to Rab8, also Rab11 is able to interplay with Rac1 and regulate cell migration [76]. Rab11 regulates transport from recycling endosomes to the plasma membrane, and it has been associated, together with Rac1, to colorectal carcinoma and cervical cancer, where it regulates tumor progression and metastasis through two different processes, collective cell migration and hypoxia [76,77,95]. Collective cell migration has been reported to occur during invasion in malignancy. Indeed, expression of Rab11 and its interactor E-cadherin have been associated with poor survival in patients affected by colorectal cancer [76]. Moreover, by interacting with the adhesion molecule E-cadherin, Rab11 is able to promote cell-cell contacts and increase Rac1 activity and expression of the matrix metalloproteinase-2. All these effects contribute to the increased collective cell migration, Rab11-dependent collective cell invasion and anchorage-independent cell growth [76]. Therefore, Rab11 activity could be important in the early stages of colorectal cancer. 

A condition that stimulates cancer invasion and migration is hypoxia. Hypoxia can occur in many solid tumors where there is poor vascularization. Therefore, hypoxia functions as selective pressure for the survival of the most aggressive and metastatic cells and could promote tumor cell invasion and lead to poor prognosis and low survival rate for the patient. Rab11 has been recently associated with hypoxia-stimulated invasion and migration of cervical cancer cells [77]. In fact, Rab11 stimulates αvβ3 integrin and the activation of FAK and PI3K through their phosphorylation under hypoxia, which then affect the expression and localization of Rac1. Indeed, when Rab11 is present, Rac1 is more expressed in hypoxia, and it is distributed also in plasma membrane and cytoplasm, besides the nucleus, where it normally localizes during normoxia [77]. Therefore, Rab11 plays an important role in coordination with Rac1 for the regulation of cell migration and development of metastasis through its action in hypoxia and collective cell migration [76,77] (Figure 1b).

Another Rab GTPase, called Rab23, is important during mouse development, and it has been associated with several types of cancer. Interestingly, Rab23 was found to be over-expressed in squamous cell carcinoma cells, and it was demonstrated that active Rab23 interacts with β1 integrin and, through it, with Tiam1, which mediates Rac1 activation and cell migration and invasion [58] (Figure 1e). Moreover, Rab23 was found absent in normal astrocytes, while it was expressed in almost 50% of astrocytoma-affected patients, showing a correlation with a higher stage of cancer progression [78]. Thus, it was demonstrated that Rab23 modulates Rac1 activity and, in turn, cell proliferation, colony formation, migration, and invasion [78]. 

A strong connection between Rab35 protein and Rac1 has been proven [79]. In fact, Wnt5a, which is normally involved in cell growth, proliferation, differentiation, motility, and survival, activates Disheveled 2 (Dvl2) by phosphorylating it, and Dvl2 then interacts with Rab35. Subsequently, Rab35 activates Rac1, which, in turn, promotes cell migration of breast cancer cells [79] (Figure 1d). 

All together, the studies conducted on the coordination of Rac1 and Rab proteins in several processes occurring during cancer development, progression, and metastasis show how interconnected the roles of these proteins are (Figure 1). Interestingly, research on the use of Rac1 inhibitors in the case of common and/or aggressive tumors showed that Rac1 could be a good target for counteracting cancer progression [58,78].

## 4. Rac1 in Neurological Diseases: Role of Rab Proteins

Rac1 plays an important role in the central and peripheral nervous systems, as it regulates neuronal migration and axon development, which are related to brain development and regeneration after axonal injuries. Indeed, while neuronal migration is important for the brain formation as it allows neurons to reach their final position during development, neurite outgrowth is fundamental for both brain development, through the formation of neuronal networks, and for the regeneration of injured axons [96,97,98,99,100,101]. Notably, also Rab proteins play several key roles in neurons, often in the same processes controlled by Rac1 [102,103].

A human brain contains hundreds of billions of neurons connected together in a complex network, which is responsible for all human functions including behavior, personality, and thoughts. During brain development, newborn neurons have to migrate from specific areas to other parts of the brain, where they will establish important communications with other neurons through the production of neurites, which are dendrites and axons. Rac1 is involved in neuronal migration as its inhibition or the inhibition of its regulator Tiam1 causes a strong impairment of the radial migration of neurons without affecting their differentiation [104]. The initial phase of neuronal migration is accompanied by an increase in volume of the proximal leading process, which is called proximal cytoplasmic dilation in the leading process (PCDLP). At this step, active Rac1 and actin assembly are fundamental and regulated by POSH (plenty of SH3s) [105]. In fact, disruption of Rac1 activity affects actin assembly and PCDLP, impairing neuronal migration [105]. Given the role of Rac1 in cell migration and actin reorganization, this small GTPase has been linked to several diseases associated with brain development, such as lissencephaly and intellectual disability [98,106]. Lissencephaly, literally meaning smooth brain, is a severe brain malformation characterized by a smooth or nearly smooth cerebral surface caused by abnormal neuronal migration during brain development. Lissencephaly is a genetically heterogeneous disorder, but the *LIS1* gene seems to have an important role in this disease, as the complete loss of Lis1 is lethal for the embryo, while *LIS1* haploinsufficiency alters neuronal migration through the upregulation of RhoA and the downregulation of Rac1 and Cdc42 activities [106,107]. Intellectual disability (ID) is a neurodevelopmental disorder where intellectual and adaptive functioning are severely impaired due to altered synaptic network and excitation/inhibition balance of specific cerebral areas. Hereditary forms of ID are caused by mutations in the *RAC1* gene or in genes regulating this small GTPase functionality. Mutations that occur in these genes determine a hypo- or hyper-activity of Rac1, impairing actin dynamics at the growth cone that affect the formation of dendrites and axons, leading to altered neuronal connections and ID [98]. Although in these diseases, only the involvement of GTPases of the RHO family has been demonstrated, it is tempting to speculate that a key role could be played also by Rab proteins, as also the transport mediated by Rab proteins is required for neuronal positioning [108] and considering that coordination between Rac1 and Rab proteins is fundamental for the regulation of many cellular processes.

The role of Rac1 in neurite outgrowth and extension is important also for axonal regeneration. In fact, despite the fact that axonal regeneration after nerve transection is not frequent in the central nervous system (CNS), injured axons can regenerate in the peripheral nervous system (PNS) due to the lack of inhibitory molecules and the presence of a fine coordination among cellular and molecular elements that assure an optimal microenvironment for axonal regrowth. Moreover, Rac1 regulates actin polymerization during myelination and demyelination in the PNS [97,109]. The sciatic nerve is the largest peripheral nerve considering both length and cross-sectional area. Due to its size, repairing of this nerve after injury can be complex. In order to identify the key molecules in axonal regeneration, several approaches have been tried, included an mRNA expression profile-based study in dorsal root ganglia (DRG) before and after sciatic nerve transection [97]. This study has discovered several genes with altered neuronal expression after peripheral nerve injury. Among these, the genes coding for myosin X and Fn14 are related to actin reorganization and protrusion formation. In particular, Fn14 contributes to nerve regeneration by interacting and co-localizing with Rac1 [97]. Interestingly, Fn14 seems to be specific for peripheral nerve regeneration as it is induced during this phase, but it is not expressed during neuronal development [97]. Similar to Rac1, Rab proteins seem to have a specific role after nerve injury [110,111], and therefore, a cooperation between them could be envisaged.

In neuronal disorders as well, a connection between Rac1 and Rab proteins could be determinant for a healthy or pathologic state. Rac1’s role in amyotrophic lateral sclerosis (ALS) disease seems to involve Rab5 activity [59,112]. ALS is a progressive and heterogeneous neurodegenerative disease that affects nerve cells in the brain and the spinal cord and is characterized by degeneration and death of motor neurons and loss of muscle movement. A small percentage of ALS (5–10%) can be inherited, and mutations in the *ALS2* gene, coding for the alsin protein, have been linked to a juvenile inherited form of ALS. Interestingly, alsin interacts with Rac1 and was initially thought to be a Rab5-GEF for the three Rab5 isoforms, even though the highest activity is on Rab5a [112]. Alsin colocalizes on Rab5-positive endosomes and also on Rac1-positive structures such as lamellipodia and membrane ruffles, and a partial colocalization between alsin, Rab5, and Rac1 has been detected on punctuate structures [112]. However, in contrast to previous work, it has been demonstrated that alsin is not a Rac GEF, but an effector of Rac1, preferably interacting with its active form [59,112]. Indeed, Rac1 recruits alsin to membrane ruffles and then to macropinosomes, where it is important for macropinocytosis. Subsequently, alsin regulates the fusion of these macropinosomes with transferrin-positive endosomes through the action of Rab5, regulating the maturation of the macropinosomes [59]. Therefore, alsin, in coordination with Rac1 and Rab5, seems to be important for membrane trafficking events that are fundamental for the integrity of motor neurons and that could be impaired in ALS [59,112].

Another Rab protein that could be involved, together with Rac1, in neurological diseases is Rab7a. Despite the fact that there is no direct demonstration of the coordination of these two GTPases on a specific disease, mutations of the *RAB7A* gene are responsible for the onset of Charcot-Marie-Tooth disease type 2B (CMT2B), a dominant axonal peripheral neuropathy. CMT2B is characterized by prominent large and small fiber sensory loss and distal muscle weakness and atrophy that may lead to ulcerations and amputations [113]. Retrograde axonal transport of neurotrophins and neurotrophins’ receptors and neuritogenic signaling are regulated by Rab7a in neurons, and expression of CMT2B-associated Rab7a mutant proteins inhibits neurite outgrowth [114,115,116,117]. Furthermore, in zebrafish, axonal growth and branching are reduced in neurons expressing CMT2B-associated Rab7a mutants [118]. Furthermore, it has been demonstrated that a number of Rab proteins, such as Rab5, Rab7a, and Rab11, regulate neuronal migration in concert with members of the RHO GTPase family, such as RhoA, Rac1, and Cdc42 [119,120]. As Rab7a interacts with Rac1 and together they regulate cell migration and adhesion, it would be interesting to evaluate the interaction of Rac1 with the CMT2B-associated Rab7a mutants [55,56]. In fact, Rac1 is important for neurite outgrowth, and altered interaction between Rac1 and CMT2B-associated Rab7a mutant compared to the wild-type protein could affect axonal regeneration and would explain the specificity of the effects of Rab7a mutated forms in the peripheral nervous system. Recent work proved that Rab7a mutants alter autophagy, suggesting that impairment of the autophagic flux could be one of the factors leading to neurodegeneration [121]. Interestingly, enhancement of autophagy can be achieved by inhibiting the Rac1-mTOR signaling pathway [122], suggesting again a coordination between Rab7a and Rac1.

Rab29 and Rac1 are involved in Parkinson's disease through the protein leucine-rich repeat kinase 2. Parkinson's disease is a progressive disorder of the nervous system where several areas of the brain are affected, especially the region called substantia nigra, which is responsible for balance and movement. Parkinson’s disease can be inherited, and mutations in the *LRRK2* gene, which codes for the protein leucine-rich repeat kinase 2, have been associated with this neurological pathology [123]. LRRK2 interacts with Rac1 and Rab29, showing a higher affinity for Rab29. Rab29 controls endosome-to-TGN retrograde membrane trafficking, a step of transport that is affected also by the disease-associated mutant of LRRK2. In addition, this mutant induces neurite shortening, which is counteracted by both Rac1 and Rab29, suggesting a common role of these two GTPases in the neurite outgrowth process [123].

Rab35 is involved in the regulation of endocytic receptor recycling, in cytokinesis, and in neurite outgrowth together with Rac1 [124]. In fact, Rab35 co-localizes with actin filaments and with Rac1, modulating the actin cytoskeleton. Co-localization of Rab35 and Rac1 also on macropinosomes has been detected, and it has been demonstrated that Rab35 regulates neurite outgrowth and cell shape through its action on Rho proteins [124].

## 5. Rac1 and Rab35 in Infections: Regulation of the Innate Immune Response

Humans are constantly exposed to potential pathogens from the surrounding environment that can cause dangerous infections. The ability to avoid infections depends on two types of responses from the human body: the innate immune response and the adaptive immune response [125]. While the adaptive immune response relies on the previous contact with a specific infectious agent and it is slow to develop after the first exposure to a new pathogen, the innate immune response is the first human defense and protects quickly from infections. In the innate immune system, pathogens are recognized from generic shared features, inflammatory response is activated, and phagocytic cells react to destroy the pathogen. In fact, phagocytic cells migrate to the site of infection, stretch themselves, and engulf the foreign body in a process called phagocytosis, which requires morphological changes based on the actin structure and which leads to the pathogen being trapped in a compartment called the phagosome and then killed [125].

The adaptive immune system relies on the immunological memory after an initial response to a specific pathogen and leads to an enhanced response to the same pathogen in the future. The adaptive immune response is triggered by the recognition of the pathogen by antigen-presenting cells (APCs) that will be exposed on the plasma membrane through the major histocompatibility complexes’ (MHC) specific antigens that will prompt in turn the activation of T cells [126]. Indeed, T cells will become polarized toward the APC and will form the immunological synapse at the interface between the APC and the T cell, an important step in the immune response. T cell migration is very important in order to encounter the APCs exposing the antigen [126].

The innate immune response exists both in vertebrates and invertebrates. Interestingly, intracellular survival and proliferation of *Salmonella enterica* serovar *typhimurium* relies on the modulation of nischarin, a Rac1, Rab4, Rab14, and Rab9 effector [127]. In fact, the interaction of nischarin with GTPases regulates maturation and acidification of the vacuoles containing bacteria, and its recruitment to these vacuoles facilitates the survival of intracellular bacteria [127].

In *Drosophila melanogaster*, only the innate immune system is present, and two different types of innate immune response are raised: humoral and cell-mediated [128]. While some specific pathways are activated and lead to the production of antimicrobial peptides, hemocytes, which are the phagocytes of invertebrates, internalize the pathogen in order to destroy it through maturation of the phagosome into phagolysosomes. Therefore, a rearrangement of actin cytoskeleton is important both for immune cell migration to the site of infection and for the phagocytosis of the pathogen through the rearrangement of actin filaments at the plasma membrane. Rab35 is important both for the regulation of the transport of organelles involved in phagocytosis and for the localization of Cdc42 and Rac1 at the plasma membrane where they control the formation of protrusions responsible for generating the phagocytic cup and where they colocalize with Rab35 [129]. In fact, upon loss of Rab35, higher lethality after infection was observed, whereas the Rab35 mutant flies showed defects in phagocytosis in vivo [129]. While the formation of ruffles was reduced at the plasma membrane upon Rab35 silencing, numerous large vesicles surrounded by actin polymers were observed when Rab35 was depleted or when the negative mutant of Rab35 was expressed [129]. Furthermore, the transport of Rac1- and Cdc42-containing vesicles by Rab35 occurred through microtubule tracks [129]. Therefore, as Rab35 is a key regulator of phagocytosis by controlling Rac1 and Cdc42 transport to the plasma membrane and actin structure remodelling, alteration of the Rab-Rho coordination could be responsible for higher susceptibility to infections.

## 6. Possible Roles of Rac1 and Rab Proteins in Other Diseases

Rac1 and Rab proteins’ coordination has been proven to be important for mechanisms involved in cancer, neurological diseases, and infections. However, other connections between Rabs and Rac1 have been identified, and these could have an important role in other pathologies. For instance, Rab7a, already linked to cell migration and to neuropathy, has been related to fundamental functions together with Rac1 also in osteoclasts and in thyroid cells [55,57]. Indeed, Rab7a is involved in the formation of the ruffled border, finger-like processes that constitute a late endosomal-like compartment at the bone-facing plasma membrane of bone resorbing osteoclasts. This structure increases the surface area and facilitates delivery of digestive enzymes for bone degradation. Therefore, Rab7a functions in the bone resorption by interacting and colocalizing with Rac1 at the fusion zone of the ruffled border and regulating the rapid fusion of intracellular acidic vesicles to the plasma membrane, possibly mediating, through the cooperation with Rac1, the transport of these vesicles between microtubules and actin microfilaments, as no microtubules reach the peripheral ruffled border [55]. Downregulation of Rab7a prevents the formation of the ruffled border and bone resorption [130], and therefore, alterations of the interaction between Rab7a and Rac1 could result in a pathological state. In fact, bone resorption is fundamental for skeletal modeling during growth and for bone remodeling of the skeleton in adults, and impairment of this osteoclast function has been already related to the tissue destruction in psoriatic arthritis and rheumatological disorders, but also to Paget’s disease of bone (PDB), a chronic and focal bone disorder, to osteoporosis, and to bone changes secondary to cancer [131,132,133,134].

Both Rab7a and Rac1 have been associated with the formation of circular dorsal ruffles (CDRs), dynamic protrusions containing actin structures located in apical surfaces of cells that might be important for internalization of molecules and cell motility. In particular, overexpression of Rab7a could induce the formation of CDRs by interacting and modulating the activity of Rac1, which, in turn, regulates the actin cytoskeleton and the genesis of CDRs. Moreover, Rab7a partially colocalizes with these large structures when overexpressed [57]. As Rab7a overexpressing cells are able to internalize thyroglobulin through the CDRs and in thyroid autonomous adenomas, Rab7a is upregulated in order to promote thyroglobulin endocytosis, it is possible that the higher thyroglobulin endocytosis observed in this pathology may occur through the activation of Rac1 and the formation of CDRs [57,87]. Therefore, alteration of this axis could be involved in the onset of this type of benign tumor. 

Interestingly, it has been discovered that both Rab5a and Rab11 can regulate T cell functions together with Rac1 in the activation of the adaptive immune response [135,136]. Indeed, Rab5a is phosphorylated by protein kinase Cε (PKCε) upon integrin or chemokine stimulation. Moreover, PKCε interacts with Rab5a in migrating T cells and regulates its localization and, in turn, Rac1 activation, actin rearrangement, and T cell motility [136]. Rab11 regulates Rac1 subcellular localization and actin dynamics through its effector FIP3. This leads to impairments in the T cell spreading, immunological synapse formation, and T cell activation. Interestingly, lamellipodia formation in T cells is dependent on Rac1 localization [135]. Alteration of T cell migration and activation could be important for the functionality of the immune system, and studying the role of Rac1 and Rabs could further elucidate the molecular mechanisms of pathologies related to inappropriate immune responses.

## 7. Conclusions

Plenty of data suggest a close relationship between Rho and Rab proteins prompting the investigation of coordinated functions in order to better understand, at the molecular level, how these proteins control together a number of cellular events.

**Figure 2:**
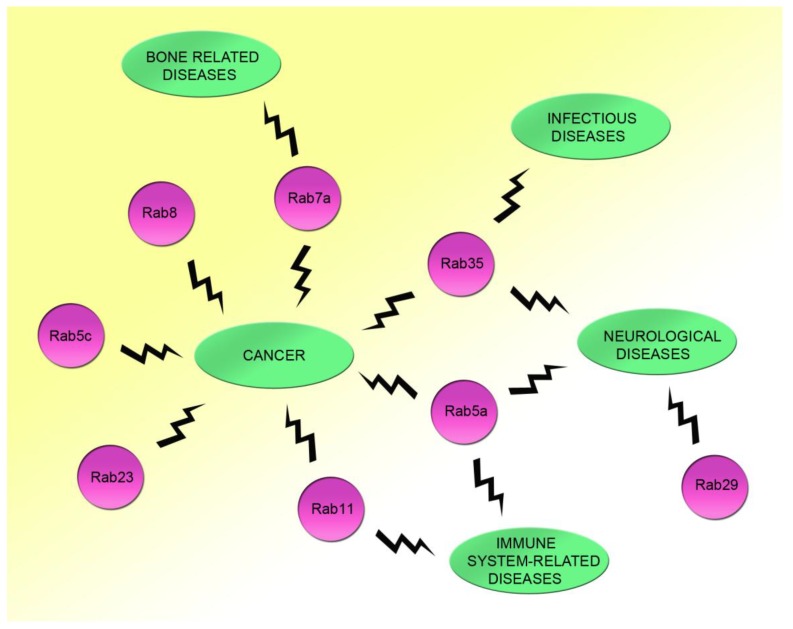
Demonstrated relationships between Rac1 and Rab proteins, whose alterations are responsible for human diseases. The interactions of Rab5c, Rab23, and Rab8 with Rac1 are important for cell migration and cancer. The coordination between Rab11 and Rac1 has a key role in immune system regulation and cancer. Rab5a and Rac1 interaction could be relevant for immune system diseases, neurological pathologies, and tumors. Rab29 and Rac1 contribute together to the onset of neurological diseases, whereas Rac1 and Rab35 interaction is altered in infectious diseases, neurological pathologies, and cancer. Finally, Rab7a has a key role in cell migration and adhesion together with Rac1, and alterations of the Rac1-Rab7a interaction could be important for bone-related diseases.

Rac1, a member of the Rho GTPases, controls the actin cytoskeleton and signal transduction, while Rab GTPases are important for the regulation of membrane trafficking. From the numerous data reported in this review, it is clear that Rac1 acts often in combination with Rab proteins, indicating that, in general, regulation of actin cytoskeleton by Rac1 and regulation of vesicular transport steps by Rab proteins are finely-coordinated events important for controlling and modulating numerous cellular functions. Indeed, coordination is emerging as a central mechanism to ensure precise control of membrane trafficking and, in turn, of multiple cellular functions. Notably, impairment of this coordination leads to several kinds of human diseases, ranging from cancer to infectious and/or neurodegenerative diseases (Figure 2). Further investigation on this coordination could help to elucidate the molecular mechanisms underlying a number of important human diseases, opening the way to the identification of targets for future effective therapeutical means.

## Figures and Tables

**Table 1:** Post-translational modifications of Rac1.

Type of Modification	Chemical Group Added	Aminoacidic Residue	Effect	Reference
Prenylation	Prenyl group	Cys178	Rac1 localization and activity	[2]
Palmitoylation	Palmitoyl group			
Ubiquitination	Ubiquitin	Lys147	Rac1 degradation and regulation of its activity	[4,5,9]
SUMOylation	Small Ubiquitin-related Modifier (SUMO) proteins	Lysine residues; C-terminal domain	Rac1 activity and cell migration	[6]
Phosphorylation	Phosphate group	Ser71	Rac1 GTP-binding activity	[7]
Phosphorylation	Phosphate group	Thr108	Rac1 activity, interaction with PLC-γ1 and cell migration	[8]

## References

[1] Haga R.B., Ridley A.J. (2016). Rho gtpases: Regulation and roles in cancer cell biology. Small GTPases.

[2] Navarro-Lérida I., Sánchez-Perales S., Calvo M., Rentero C., Zheng Y., Enrich C., Del Pozo M.A. (2012). A palmitoylation switch mechanism regulates rac1 function and membrane organization. EMBO J..

[3] Lynch E.A., Stall J., Schmidt G., Chavrier P., D'Souza-Schorey C. (2006). Proteasome-mediated degradation of rac1-gtp during epithelial cell scattering. Mol. Biol. Cell.

[4] Visvikis O., Lorès P., Boyer L., Chardin P., Lemichez E., Gacon G. (2008). Activated rac1, but not the tumorigenic variant rac1b, is ubiquitinated on lys 147 through a jnk-regulated process. FEBS J..

[5] Torrino S., Visvikis O., Doye A., Boyer L., Stefani C., Munro P., Bertoglio J., Gacon G., Mettouchi A., Lemichez E. (2011). The e3 ubiquitin-ligase hace1 catalyzes the ubiquitylation of active rac1. Dev. Cell.

[6] Castillo-Lluva S., Tatham M.H., Jones R.C., Jaffray E.G., Edmondson R.D., Hay R.T., Malliri A. (2010). Sumoylation of the gtpase rac1 is required for optimal cell migration. Nat. Cell Biol..

[7] Kwon T., Kwon D.Y., Chun J., Kim J.H., Kang S.S. (2000). Akt protein kinase inhibits rac1-gtp binding through phosphorylation at serine 71 of rac1. J. Biol. Chem..

[8] Tong J., Li L., Ballermann B., Wang Z. (2013). Phosphorylation of rac1 t108 by extracellular signal-regulated kinase in response to epidermal growth factor: A novel mechanism to regulate rac1 function. Mol. Cell Biol..

[9] Castillo-Lluva S., Tan C.T., Daugaard M., Sorensen P.H., Malliri A. (2013). The tumour suppressor hace1 controls cell migration by regulating rac1 degradation. Oncogene.

[10] Schwarz J., Proff J., Hävemeier A., Ladwein M., Rottner K., Barlag B., Pich A., Tatge H., Just I., Gerhard R. (2012). Serine-71 phosphorylation of rac1 modulates downstream signaling. PLoS ONE.

[11] Osborn-Heaford H.L., Ryan A.J., Murthy S., Racila A.M., He C., Sieren J.C., Spitz D.R., Carter A.B. (2012). Mitochondrial rac1 gtpase import and electron transfer from cytochrome c are required for pulmonary fibrosis. J. Biol. Chem..

[12] Murthy S., Ryan A., He C., Mallampalli R.K., Carter A.B. (2010). Rac1-mediated mitochondrial h2o2 generation regulates mmp-9 gene expression in macrophages via inhibition of sp-1 and ap-1. J. Biol. Chem..

[13] Marei H., Carpy A., Macek B., Malliri A. (2016). Proteomic analysis of rac1 signaling regulation by guanine nucleotide exchange factors. Cell Cycle.

[14] Quinn C.C., Pfeil D.S., Wadsworth W.G. (2008). Ced-10/rac1 mediates axon guidance by regulating the asymmetric distribution of mig-10/lamellipodin. Curr. Biol..

[15] Kärkkäinen S., van der Linden M., Renkema G.H. (2010). Posh2 is a ring finger e3 ligase with rac1 binding activity through a partial crib domain. FEBS Lett..

[16] Suzuki T., Mimuro H., Miki H., Takenawa T., Sasaki T., Nakanishi H., Takai Y., Sasakawa C. (2000). Rho family gtpase cdc42 is essential for the actin-based motility of shigella in mammalian cells. J. Exp. Med..

[17] Frasa M.A., Maximiano F.C., Smolarczyk K., Francis R.E., Betson M.E., Lozano E., Goldenring J., Seabra M.C., Rak A., Ahmadian M.R. (2010). Armus is a rac1 effector that inactivates rab7 and regulates e-cadherin degradation. Curr. Biol..

[18] Jezyk M.R., Snyder J.T., Gershberg S., Worthylake D.K., Harden T.K., Sondek J. (2006). Crystal structure of rac1 bound to its effector phospholipase c-beta2. Nat. Struct. Mol. Biol..

[19] Ridley A.J., Paterson H.F., Johnston C.L., Diekmann D., Hall A. (1992). The small gtp-binding protein rac regulates growth factor-induced membrane ruffling. Cell.

[20] Guo F., Debidda M., Yang L., Williams D.A., Zheng Y. (2006). Genetic deletion of rac1 gtpase reveals its critical role in actin stress fiber formation and focal adhesion complex assembly. J. Biol. Chem..

[21] Chung C.Y., Lee S., Briscoe C., Ellsworth C., Firtel R.A. (2000). Role of rac in controlling the actin cytoskeleton and chemotaxis in motile cells. Proc. Natl. Acad. Sci. USA.

[22] Verma S.K., Lal H., Golden H.B., Gerilechaogetu F., Smith M., Guleria R.S., Foster D.M., Lu G., Dostal D.E. (2011). Rac1 and rhoa differentially regulate angiotensinogen gene expression in stretched cardiac fibroblasts. Cardiovasc. Res..

[23] Hill C.S., Wynne J., Treisman R. (1995). The rho family gtpases rhoa, rac1, and cdc42hs regulate transcriptional activation by srf. Cell.

[24] Westwick J.K., Lambert Q.T., Clark G.J., Symons M., Van Aelst L., Pestell R.G., Der C.J. (1997). Rac regulation of transformation, gene expression, and actin organization by multiple, pak-independent pathways. Mol. Cell Biol..

[25] Giehl K., Keller C., Muehlich S., Goppelt-Struebe M. (2015). Actin-mediated gene expression depends on rhoa and rac1 signaling in proximal tubular epithelial cells. PLoS ONE.

[26] Santibáñez J.F., Kocić J., Fabra A., Cano A., Quintanilla M. (2010). Rac1 modulates tgf-beta1-mediated epithelial cell plasticity and mmp9 production in transformed keratinocytes. FEBS Lett..

[27] Salvatierra E., Alvarez M.J., Leishman C.C., Rivas Baquero E., Lutzky V.P., Chuluyan H.E., Podhajcer O.L. (2015). Sparc controls melanoma cell plasticity through rac1. PLoS ONE.

[28] Moldovan L., Irani K., Moldovan N.I., Finkel T., Goldschmidt-Clermont P.J. (1999). The actin cytoskeleton reorganization induced by rac1 requires the production of superoxide. Antioxid. Redox Sign..

[29] Li S.M., Zeng L.W., Feng L., Chen D.B. (2010). Rac1-dependent intracellular superoxide formation mediates vascular endothelial growth factor-induced placental angiogenesis in vitro. Endocrinology.

[30] Joneson T., Bar-Sagi D. (1998). A rac1 effector site controlling mitogenesis through superoxide production. J. Biol. Chem..

[31] Doanes A.M., Irani K., Goldschmidt-Clermont P.J., Finkel T. (1998). A requirement for rac1 in the pdgf-stimulated migration of fibroblasts and vascular smooth cells. BioChem. Mol. Biol. Int..

[32] Keely P.J., Westwick J.K., Whitehead I.P., Der C.J., Parise L.V. (1997). Cdc42 and rac1 induce integrin-mediated cell motility and invasiveness through pi(3)k. Nature.

[33] Takaishi K., Sasaki T., Kotani H., Nishioka H., Takai Y. (1997). Regulation of cell-cell adhesion by rac and rho small g proteins in mdck cells. J. Cell Biol..

[34] Dumontier M., Höcht P., Mintert U., Faix J. (2000). Rac1 gtpases control filopodia formation, cell motility, endocytosis, cytokinesis and development in dictyostelium. J. Cell Sci..

[35] Moore K.A., Sethi R., Doanes A.M., Johnson T.M., Pracyk J.B., Kirby M., Irani K., Goldschmidt-Clermont P.J., Finkel T. (1997). Rac1 is required for cell proliferation and g2/m progression. Biochem. J..

[36] Jeong H.G., Cho H.J., Chang I.Y., Yoon S.P., Jeon Y.J., Chung M.H., You H.J. (2002). Rac1 prevents cisplatin-induced apoptosis through down-regulation of p38 activation in nih3t3 cells. FEBS Lett..

[37] Zhang B., Zhang Y., Shacter E. (2004). Rac1 inhibits apoptosis in human lymphoma cells by stimulating bad phosphorylation on ser-75. Mol. Cell Biol..

[38] Muise A.M., Walters T., Xu W., Shen-Tu G., Guo C.H., Fattouh R., Lam G.Y., Wolters V.M., Bennitz J., van Limbergen J. (2011). Single nucleotide polymorphisms that increase expression of the guanosine triphosphatase rac1 are associated with ulcerative colitis. Gastroenterology.

[39] Nagase M., Kurihara H., Aiba A., Young M.J., Sakai T. (2016). Deletion of rac1gtpase in the myeloid lineage protects against inflammation-mediated kidney injury in mice. PLoS ONE.

[40] Cuadrado A., Martín-Moldes Z., Ye J., Lastres-Becker I. (2014). Transcription factors nrf2 and nf-κb are coordinated effectors of the rho family, gtp-binding protein rac1 during inflammation. J. Biol. Chem..

[41] Armstrong A.W., Makino T., Davidson M., Starcevic D., Kislat A., Nguyen N.T., Hashimoto T., Homey B., Khavari P.A., Bradley M. (2016). Rac1 activation drives pathologic interactions between the epidermis and immune cells. J. Clin. Invest..

[42] Heid I., Lubeseder-Martellato C., Sipos B., Mazur P.K., Lesina M., Schmid R.M., Siveke J.T. (2011). Early requirement of rac1 in a mouse model of pancreatic cancer. Gastroenterology.

[43] Tian Y., Xu L., He Y., Xu X., Li K., Ma Y., Gao Y., Wei D., Wei L. (2018). Knockdown of rac1 and vasp gene expression inhibits breast cancer cell migration. Oncol Lett..

[44] Yoon C., Cho S.J., Chang K.K., Park D.J., Ryeom S.W., Yoon S.S. (2017). Role of rac1 pathway in epithelial-to-mesenchymal transition and cancer stem-like cell phenotypes in gastric adenocarcinoma. Mol. Cancer Res..

[45] Ferri N., Contini A., Bernini S.K., Corsini A. (2013). Role of small gtpase protein rac1 in cardiovascular diseases: Development of new selective pharmacological inhibitors. J. Cardiovasc. Pharmacol..

[46] Zhu S., Dai J., Liu H., Cong X., Chen Y., Wu Y., Hu H., Heng B.C., Ouyang H.W., Zhou Y. (2015). Down-regulation of rac gtpase-activating protein ocrl1 causes aberrant activation of rac1 in osteoarthritis development. Arthritis. Rheumatol..

[47] Shibata S., Mu S., Kawarazaki H., Muraoka K., Ishizawa K., Yoshida S., Kawarazaki W., Takeuchi M., Ayuzawa N., Miyoshi J. (2011). Rac1 gtpase in rodent kidneys is essential for salt-sensitive hypertension via a mineralocorticoid receptor-dependent pathway. J. Clin. Invest..

[48] Shibata S., Nagase M., Yoshida S., Kawarazaki W., Kurihara H., Tanaka H., Miyoshi J., Takai Y., Fujita T. (2008). Modification of mineralocorticoid receptor function by rac1 gtpase: Implication in proteinuric kidney disease. Nat. Med..

[49] Kawarazaki W., Nagase M., Yoshida S., Takeuchi M., Ishizawa K., Ayuzawa N., Ueda K., Fujita T. (2012). Angiotensin ii- and salt-induced kidney injury through rac1-mediated mineralocorticoid receptor activation. J. Am. Soc. Nephrol..

[50] Lim J.S., Shin M., Kim H.J., Kim K.S., Choy H.E., Cho K.A. (2014). Caveolin-1 mediates salmonella invasion via the regulation of sope-dependent rac1 activation and actin reorganization. J. Infect. Dis..

[51] Criss A.K., Ahlgren D.M., Jou T.S., McCormick B.A., Casanova J.E. (2001). The gtpase rac1 selectively regulates salmonella invasion at the apical plasma membrane of polarized epithelial cells. J. Cell Sci..

[52] Stankiewicz T.R., Linseman D.A. (2014). Rho family gtpases: Key players in neuronal development, neuronal survival, and neurodegeneration. Front Cell Neurosci..

[53] Zhang X., Gao N. (2016). Rab and rho gtpases regulate intestinal crypt cell homeostasis and enterocyte function. Small GTPases.

[54] Imamura H., Takaishi K., Nakano K., Kodama A., Oishi H., Shiozaki H., Monden M., Sasaki T., Takai Y. (1998). Rho and rab small g proteins coordinately reorganize stress fibers and focal adhesions in mdck cells. Mol. Biol. Cell.

[55] Sun Y., Buki K.G., Ettala O., Vaaraniemi J.P., Vaananen H.K. (2005). Possible role of direct rac1-rab7 interaction in ruffled border formation of osteoclasts. J. Biol. Chem..

[56] Margiotta A., Progida C., Bakke O., Bucci C. (2017). Rab7a regulates cell migration through rac1 and vimentin. Biochim. Biophys. Acta.

[57] Mascia A., Gentile F., Izzo A., Mollo N., De Luca M., Bucci C., Nitsch L., Calì G. (2016). Rab7 regulates cdh1 endocytosis, circular dorsal ruffles genesis and thyroglobulin internalization in a thyroid cell line. J. Cell Physiol..

[58] Jian Q., Miao Y., Tang L., Huang M., Yang Y., Ba W., Liu Y., Chi S., Li C. (2016). Rab23 promotes squamous cell carcinoma cell migration and invasion via integrin beta1/rac1 pathway. Oncotarget.

[59] Kunita R., Otomo A., Mizumura H., Suzuki-Utsunomiya K., Hadano S., Ikeda J.E. (2007). The rab5 activator als2/alsin acts as a novel rac1 effector through rac1-activated endocytosis. J. Biol. Chem..

[60] Morrison Joly M., Williams M.M., Hicks D.J., Jones B., Sanchez V., Young C.D., Sarbassov D.D., Muller W.J., Brantley-Sieders D., Cook R.S. (2017). Two distinct mtorc2-dependent pathways converge on rac1 to drive breast cancer metastasis. Breast Cancer Res..

[61] Hong M., Zhang Z., Chen Q., Lu Y., Zhang J., Lin C., Zhang F., Zhang W., Li X., Zhang W. (2019). Irf1 inhibits the proliferation and metastasis of colorectal cancer by suppressing the ras-rac1 pathway. Cancer Manag. Res..

[62] Peng J.X., Liang S.Y., Li L. (2019). Sfrp1 exerts effects on gastric cancer cells through gsk3beta/rac1mediated restraint of tgfbeta/smad3 signaling. Oncol. Rep..

[63] Caggia S., Chunduri H., Millena A.C., Perkins J.N., Venugopal S.V., Vo B.T., Li C., Tu Y., Khan S.A. (2018). Novel role of gialpha2 in cell migration: Downstream of pi3-kinase-akt and rac1 in prostate cancer cells. J. Cell Physiol..

[64] Hu J., Meng Y., Zeng J., Zeng B., Jiang X. (2018). Ubiquitin e3 ligase march7 promotes proliferation and invasion of cervical cancer cells through vav2-rac1-cdc42 pathway. Oncol. Lett..

[65] Zhou K., Rao J., Zhou Z.H., Yao X.H., Wu F., Yang J., Yang L., Zhang X., Cui Y.H., Bian X.W. (2018). Rac1-gtp promotes epithelial-mesenchymal transition and invasion of colorectal cancer by activation of stat3. Lab. Invest..

[66] Jamieson C., Lui C., Brocardo M.G., Martino-Echarri E., Henderson B.R. (2015). Rac1 augments wnt signaling by stimulating beta-catenin-lymphoid enhancer factor-1 complex assembly independent of beta-catenin nuclear import. J. Cell Sci..

[67] Bopp A., Wartlick F., Henninger C., Schwarz M., Kaina B., Fritz G. (2015). Rac1 promotes diethylnitrosamine (den)-induced formation of liver tumors. Carcinogenesis.

[68] Ma J., Xue Y., Liu W., Yue C., Bi F., Xu J., Zhang J., Li Y., Zhong C., Chen Y. (2013). Role of activated rac1/cdc42 in mediating endothelial cell proliferation and tumor angiogenesis in breast cancer. PLoS ONE.

[69] Liang Y., Wang S., Zhang Y. (2018). Downregulation of dock1 and elmo1 suppresses the migration and invasion of triple-negative breast cancer epithelial cells through the rhoa/rac1 pathway. Oncol. Lett..

[70] Recouvreux M.V., Commisso C. (2017). Macropinocytosis: A metabolic adaptation to nutrient stress in cancer. Front Endocrinol. (Lausanne).

[71] Seyfried T.N., Huysentruyt L.C. (2013). On the origin of cancer metastasis. Crit. Rev. Oncog..

[72] Vicente-Manzanares M., Webb D.J., Horwitz A.R. (2005). Cell migration at a glance. J. Cell Sci.

[73] Liu S.S., Chen X.M., Zheng H.X., Shi S.L., Li Y. (2011). Knockdown of rab5a expression decreases cancer cell motility and invasion through integrin-mediated signaling pathway. J. Biomed. Sci..

[74] Chen P.I., Schauer K., Kong C., Harding A.R., Goud B., Stahl P.D. (2014). Rab5 isoforms orchestrate a "division of labor" in the endocytic network; rab5c modulates rac-mediated cell motility. PLoS ONE.

[75] Bravo-Cordero J.J., Cordani M., Soriano S.F., Diez B., Munoz-Agudo C., Casanova-Acebes M., Boullosa C., Guadamillas M.C., Ezkurdia I., Gonzalez-Pisano D. (2016). A novel high-content analysis tool reveals rab8-driven cytoskeletal reorganization through rho gtpases, calpain and mt1-mmp. J. Cell Sci..

[76] Chung Y.C., Wei W.C., Hung C.N., Kuo J.F., Hsu C.P., Chang K.J., Chao W.T. (2016). Rab11 collaborates e-cadherin to promote collective cell migration and indicates a poor prognosis in colorectal carcinoma. Eur J. Clin. Invest..

[77] Xu H., Yuan Y., Wu W., Zhou M., Jiang Q., Niu L., Ji J., Liu N., Zhang L., Wang X. (2017). Hypoxia stimulates invasion and migration of human cervical cancer cell lines hela/siha through the rab11 trafficking of integrin alphavbeta3/fak/pi3k pathway-mediated rac1 activation. J. Biosci..

[78] Wang M., Dong Q., Wang Y. (2016). Rab23 is overexpressed in human astrocytoma and promotes cell migration and invasion through regulation of rac1. Tumour Biol..

[79] Zhu Y., Shen T., Liu J., Zheng J., Zhang Y., Xu R., Sun C., Du J., Chen Y., Gu L. (2013). Rab35 is required for wnt5a/dvl2-induced rac1 activation and cell migration in mcf-7 breast cancer cells. Cell Signal.

[80] Bucci C., Parton R.G., Mather I.H., Stunnenberg H., Simons K., Hoflack B., Zerial M. (1992). The small gtpase rab5 functions as a regulatory factor in the early endocytic pathway. Cell.

[81] Bucci C., Wandinger-Ness A., Lutcke A., Chiariello M., Bruni C., Zerial M. (1994). Rab5a is a common component of the apical and basolateral endocytic machinery in polarized epithelial cells. Proc. Natl. Acad. Sci. USA..

[82] Sandri C., Caccavari F., Valdembri D., Camillo C., Veltel S., Santambrogio M., Lanzetti L., Bussolino F., Ivaska J., Serini G. (2012). The r-ras/rin2/rab5 complex controls endothelial cell adhesion and morphogenesis via active integrin endocytosis and rac signaling. Cell Res..

[83] Pfeffer S.R. (2013). Rab gtpase regulation of membrane identity. Curr. Opin. Cell Biol..

[84] Zhang M., Chen L., Wang S., Wang T. (2009). Rab7: Roles in membrane trafficking and disease. Biosci. Rep..

[85] Guerra F., Bucci C. (2016). Multiple roles of the small gtpase rab7. Cells.

[86] Zhou Y., Toth M., Hamman M.S., Monahan S.J., Lodge P.A., Boynton A.L., Salgaller M.L. (2002). Serological cloning of paris-1: A new tbc domain-containing, immunogenic tumor antigen from a prostate cancer cell line. Biochem. Biophys. Res. Commun..

[87] Croizet-Berger K., Daumerie C., Couvreur M., Courtoy P.J., van den Hove M.F. (2002). The endocytic catalysts, rab5a and rab7, are tandem regulators of thyroid hormone production. Proc. Natl. Acad. Sci. USA.

[88] Cantalupo G., Alifano P., Roberti V., Bruni C.B., Bucci C. (2001). Rab-interacting lysosomal protein (rilp): The rab7 effector required for transport to lysosomes. EMBO J..

[89] Margiotta A., Progida C., Bakke O., Bucci C. (2017). Characterization of the role of rilp in cell migration. Eur. J. Histochem..

[90] Huber L.A., Pimplikar S., Parton R.G., Virta H., Zerial M., Simons K. (1993). Rab8, a small gtpase involved in vesicular traffic between the tgn and the basolateral plasma membrane. J. Cell Biol..

[91] Hattula K., Furuhjelm J., Arffman A., Peranen J. (2002). A rab8-specific gdp/gtp exchange factor is involved in actin remodeling and polarized membrane transport. Mol. Biol. Cell.

[92] Peranen J., Auvinen P., Virta H., Wepf R., Simons K. (1996). Rab8 promotes polarized membrane transport through reorganization of actin and microtubules in fibroblasts. J. Cell Biol..

[93] Palamidessi A., Frittoli E., Garré M., Faretta M., Mione M., Testa I., Diaspro A., Lanzetti L., Scita G., Di Fiore P.P. (2008). Endocytic trafficking of rac is required for the spatial restriction of signaling in cell migration. Cell.

[94] Bryant D.M., Datta A., Rodriguez-Fraticelli A.E., Peranen J., Martin-Belmonte F., Mostov K.E. (2010). A molecular network for de novo generation of the apical surface and lumen. Nat. Cell Biol..

[95] Ramel D., Wang X., Laflamme C., Montell D.J., Emery G. (2013). Rab11 regulates cell-cell communication during collective cell movements. Nat. Cell Biol..

[96] Fan C., Wang J., Tang Y., Wang Y., Xiong F., Zhang S., Li X., Xiang B., Wu X., Guo C. (2018). Long non-coding rna loc284454 promotes migration and invasion of nasopharyngeal carcinoma via modulating the rho/rac signaling pathway. Carcinogenesis.

[97] Tanabe K., Bonilla I., Winkles J.A., Strittmatter S.M. (2003). Fibroblast growth factor-inducible-14 is induced in axotomized neurons and promotes neurite outgrowth. J. Neurosci..

[98] Zamboni V., Armentano M., Berto G., Ciraolo E., Ghigo A., Garzotto D., Umbach A., DiCunto F., Parmigiani E., Boido M. (2018). Hyperactivity of rac1-gtpase pathway impairs neuritogenesis of cortical neurons by altering actin dynamics. Sci. Rep..

[99] Kaufmann N., Wills Z.P., Van Vactor D. (1998). Drosophila rac1 controls motor axon guidance. Development.

[100] Aoki K., Nakamura T., Matsuda M. (2004). Spatio-temporal regulation of rac1 and cdc42 activity during nerve growth factor-induced neurite outgrowth in pc12 cells. J. Biol. Chem..

[101] Woo S., Gomez T.M. (2006). Rac1 and rhoa promote neurite outgrowth through formation and stabilization of growth cone point contacts. J. Neurosci..

[102] Bucci C., Alifano P., Cogli L. (2014). The role of rab proteins in neuronal cells and in the trafficking of neurotrophin receptors. Membranes.

[103] Kiral F.R., Kohrs F.E., Jin E.J., Hiesinger P.R. (2018). Rab gtpases and membrane trafficking in neurodegeneration. Curr. Biol..

[104] Kawauchi T., Chihama K., Nabeshima Y., Hoshino M. (2003). The in vivo roles of stef/tiam1, rac1 and jnk in cortical neuronal migration. EMBO J..

[105] Yang T., Sun Y., Zhang F., Zhu Y., Shi L., Li H., Xu Z. (2012). Posh localizes activated rac1 to control the formation of cytoplasmic dilation of the leading process and neuronal migration. Cell Rep..

[106] Kholmanskikh S.S., Dobrin J.S., Wynshaw-Boris A., Letourneau P.C., Ross M.E. (2003). Disregulated rhogtpases and actin cytoskeleton contribute to the migration defect in lis1-deficient neurons. J. Neurosci..

[107] Hirotsune S., Fleck M.W., Gambello M.J., Bix G.J., Chen A., Clark G.D., Ledbetter D.H., McBain C.J., Wynshaw-Boris A. (1998). Graded reduction of pafah1b1 (lis1) activity results in neuronal migration defects and early embryonic lethality. Nat. Genet..

[108] Houalla T., Shi L., van Meyel D.J., Rao Y. (2010). Rab-mediated vesicular transport is required for neuronal positioning in the developing drosophila visual system. Mol. Brain.

[109] Park H.T., Feltri M.L. (2011). Rac1 gtpase controls myelination and demyelination. Bioarchitecture.

[110] Casey L., Riyadh M.A., Yan Ho X., Neumann B., Giordano-Santini R., Hilliard M.A. (2019). Disruption of rab-5 increases eff-1 fusogen availability at the cell surface and promotes the regenerative axonal fusion capacity of the neuron. J. Neurosci..

[111] Egami Y., Kiryu-Seo S., Yoshimori T., Kiyama H. (2005). Induced expressions of rab24 gtpase and lc3 in nerve-injured motor neurons. Biochem. Biophys. Res. Commun..

[112] Topp J.D., Gray N.W., Gerard R.D., Horazdovsky B.F. (2004). Alsin is a rab5 and rac1 guanine nucleotide exchange factor. J. Biol. Chem..

[113] Bucci C., De Luca M. (2012). Molecular basis of charcot-marie-tooth type 2b disease. Biochem. Soc. Trans..

[114] Deinhardt K., Salinas S., Verastegui C., Watson R., Worth D., Hanrahan S., Bucci C., Schiavo G. (2006). Rab5 and rab7 control endocytic sorting along the axonal retrograde transport pathway. Neuron.

[115] Saxena S., Bucci C., Weis J., Kruttgen A. (2005). The small gtpase rab7 controls the endosomal trafficking and neuritogenic signaling of the nerve growth factor receptor trka. J. Neurosci..

[116] Cogli L., Progida C., Lecci R., Bramato R., Krüttgen A., Bucci C. (2010). Cmt2b-associated rab7 mutants inhibit neurite outgrowth. Acta Neuropathol..

[117] Yamauchi J., Torii T., Kusakawa S., Sanbe A., Nakamura K., Takashima S., Hamasaki H., Kawaguchi S., Miyamoto Y., Tanoue A. (2010). The mood stabilizer valproic acid improves defective neurite formation caused by charcot-marie-tooth disease-associated mutant rab7 through the jnk signaling pathway. J. Neurosci. Res..

[118] Ponomareva O.Y., Eliceiri K.W., Halloran M.C. (2016). Charcot-marie-tooth 2b associated rab7 mutations cause axon growth and guidance defects during vertebrate sensory neuron development. Neural. Dev..

[119] Kawauchi T., Sekine K., Shikanai M., Chihama K., Tomita K., Kubo K., Nakajima K., Nabeshima Y., Hoshino M. (2010). Rab gtpases-dependent endocytic pathways regulate neuronal migration and maturation through n-cadherin trafficking. Neuron.

[120] Kawauchi T. (2011). Regulation of cell adhesion and migration in cortical neurons: Not only rho but also rab family small gtpases. Small GTPases.

[121] Colecchia D., Stasi M., Leonardi M., Manganelli F., Nolano M., Veneziani B.M., Santoro L., Eskelinen E.-L., Chiariello M., Bucci C. (2018). Alterations of autophagy in charcot-marie-tooth type 2b. Autophagy.

[122] Wei Y.M., Li X., Xu M., Abais J.M., Chen Y., Riebling C.R., Boini K.M., Li P.L., Zhang Y. (2013). Enhancement of autophagy by simvastatin through inhibition of rac1-mtor signaling pathway in coronary arterial myocytes. Cell Physiol. Biochem..

[123] Feng M., Hu X., Li N., Hu F., Chang F., Xu H.F., Liu Y.J. (2018). Distinctive roles of rac1 and rab29 in lrrk2 mediated membrane trafficking and neurite outgrowth. J. Biomed. Res..

[124] Chevallier J., Koop C., Srivastava A., Petrie R.J., Lamarche-Vane N., Presley J.F. (2009). Rab35 regulates neurite outgrowth and cell shape. FEBS Lett..

[125] Turvey S.E., Broide D.H. (2010). Innate immunity. J. Allergy Clin. Immunol..

[126] Netea M.G., Schlitzer A., Placek K., Joosten L.A.B., Schultze J.L. (2019). Innate and adaptive immune memory: An evolutionary continuum in the host's response to pathogens. Cell Host. Microbe..

[127] Kuijl C., Pilli M., Alahari S.K., Janssen H., Khoo P.S., Ervin K.E., Calero M., Jonnalagadda S., Scheller R.H., Neefjes J. (2013). Rac and rab gtpases dual effector nischarin regulates vesicle maturation to facilitate survival of intracellular bacteria. EMBO J..

[128] Nehme N.T., Quintin J., Cho J.H., Lee J., Lafarge M.C., Kocks C., Ferrandon D. (2011). Relative roles of the cellular and humoral responses in the drosophila host defense against three gram-positive bacterial infections. PLoS ONE.

[129] Shim J., Lee S.M., Lee M.S., Yoon J., Kweon H.S., Kim Y.J. (2010). Rab35 mediates transport of cdc42 and rac1 to the plasma membrane during phagocytosis. Mol. Cell Biol..

[130] Zhao H., Laitala-Leinonen T., Parikka V., Väänänen H.K. (2001). Downregulation of small gtpase rab7 impairs osteoclast polarization and bone resorption. J. Biol. Chem..

[131] Mensah K.A., Schwarz E.M., Ritchlin C.T. (2008). Altered bone remodeling in psoriatic arthritis. Curr. Rheumatol. Rep..

[132] Gennari L., Rendina D., Falchetti A., Merlotti D. (2019). Paget's disease of bone. Calcif. Tissue Int..

[133] Ng P.Y., Brigitte Patricia Ribet A., Pavlos N.J. (2019). Membrane trafficking in osteoclasts and implications for osteoporosis. Biochem. Soc. Trans..

[134] Vallet S., Filzmoser J.M., Pecherstorfer M., Podar K. (2018). Myeloma bone disease: Update on pathogenesis and novel treatment strategies. Pharmaceutics.

[135] Bouchet J., Del Rio-Iniguez I., Lasserre R., Aguera-Gonzalez S., Cuche C., Danckaert A., McCaffrey M.W., Di Bartolo V., Alcover A. (2016). Rac1-rab11-fip3 regulatory hub coordinates vesicle traffic with actin remodeling and t-cell activation. EMBO J..

[136] Ong S.T., Freeley M., Skubis-Zegadlo J., Fazil M.H., Kelleher D., Fresser F., Baier G., Verma N.K., Long A. (2014). Phosphorylation of rab5a protein by protein kinase c is crucial for t-cell migration. J. Biol. Chem..

